# Metabolite Characteristics in Tongue Coating from Damp Phlegm Pattern in Patients with Gastric Precancerous Lesion

**DOI:** 10.1155/2021/5515325

**Published:** 2021-06-02

**Authors:** Yifeng Xu, Renling Zhang, Robert Morris, Feng Cheng, Yiqin Wang, Zhujing Zhu, Yiming Hao

**Affiliations:** ^1^Shanghai Key Laboratory of Health Identification and Assessment/Laboratory of TCM Four Diagnostic Information, Shanghai University of Traditional Chinese Medicine, Shanghai 201203, China; ^2^Longhua Hospital, Shanghai University of Traditional Chinese Medicine, Shanghai 200032, China; ^3^Department of Pharmaceutical Sciences, College of Pharmacy, University of South Florida, Tampa, FL 33612, USA

## Abstract

**Objective:**

In this study, we analyzed the metabolite profile of the tongue coating of patients having gastric precancerous lesion (GPL) with damp phlegm pattern and proposed a mechanism of pathological transition.

**Methods:**

The changes in tongue-coating metabolites in patients with GPL damp phlegm pattern were analyzed using GC-TOF-MS and UHPLC-QE-MS metabolomics methods.

**Results:**

When compared with 20 patients who did not exhibit a nondamp phlegm pattern, 12 metabolites were highly expressed and 10 metabolites were under expressed in 40 cases of damp phlegm pattern, of which involved 9 metabolic pathways. Compared with 15 healthy people, 134 metabolites were upregulated and 3 metabolites were downregulated in 40 cases exhibiting a damp phlegm pattern, of which involved 17 metabolic pathways. The patients with damp phlegm pattern were compared with nondamp phlegm pattern patients and healthy people, the main differential metabolites were primarily lipids and lipid-like molecules, and the main differential metabolic pathways were related to glycerophospholipid metabolism. In the glycerophospholipid metabolism, the metabolites with changes were phosphatidylethanolamine and lysoPC(18 : 1 (9z)). Among them, phosphatidylethanolamine exists in the synthesis stage of glycerophospholipid metabolism.

**Conclusions:**

Abnormal expression of lipids and lipid-like molecules, as the major metabolic change, was involved in the formation of GPL patients with damp phlegm pattern.

## 1. Introduction

Gastric cancer (GC) has grown in frequency and has become the third leading cause of cancer-related deaths in the world [1]. China is a region in which gastric cancer has a high prevalence with studies estimating that the number of gastric cancer cases in China alone is approximately 697,000, accounting for 70% of the total world incidence [2]. Gastric precancerous lesions (GPLs) involve a process of evolution before the onset of gastric cancer and include intestinal metaplasia and dysplasia, which are primarily related to chronic atrophic gastritis [3]. GPL has the characteristics of bidirectional transformation, and early intervention can effectively reverse the malignant development of cells [4]. Therefore, early diagnosis and early treatment of GPL play a crucial role in reducing the incidence of gastric cancer [5].

Traditional Chinese medicine (TCM) theory is the theoretical basis of damp phlegm pattern. In TCM, damp phlegm pattern is caused by dysfunction of an internal organ, resulting in disrupted body water movement and water stagnation that forms dampness and phlegm. Fullness and heaviness of the whole body as well as a greasy tongue coating are the main manifestations of damp phlegm pattern in patients. Currently, the World Health Organization has listed damp phlegm pattern in the International Classification of Diseases [6]. Similarly, phlegm dampness pattern is also one of the main patterns of GPL [7].

In TCM, tongue diagnosis as a noninvasive diagnostic method, which is very important in the identification of disease states. Tongue diagnosis is designed to observe the changes of the tongue body and tongue coating over time in order to understand the physiological state of the human body. Inspection of tongue coating is the main component of tongue diagnosis. TCM holds that the tongue coating can react very sensitively to abnormal changes in the spleen and stomach. Compared with the thin coating of physiologically healthy people, the tongue coating of chronic gastritis patients that exhibit a damp phlegm pattern is greasy. Studies have also shown that the shape of the tongue coating in gastric cancer and chronic gastritis patients differed significantly from the tongue-coating shape of patients without gastric complications [8, 9]. Tongue coating adheres to the tongue body and consists of desquamated epithelial cells, blood cells, metabolites, nutrients, and bacteria [10]. In our previous study, we found that there were differential metabolites in the tongue coating of patients with coronary heart disease and chronic renal failure with damp phlegm pattern [11]; other researchers also found differential metabolites in the tongue coating of hepatitis B patients compared with healthy people [12]. As a result, the tongue-coating metabolites obtained by the noninvasive method can be used as adjunctive diagnostic tools for some diseases [13]. However, current studies on the changes of GPL patients' tongue coating in damp phlegm pattern are still insufficient.

Metabolomics is a scientific discipline that reveals the nature of life metabolism by investigating the alterations of metabolite profiles in a biological system in response to stimulation or disturbance [14, 15]. The combination of mass spectrometry (MS) and nuclear magnetic resonance (NMR) and other separation methods have become important analytical tools in metabolomics [16].

Some researchers have used ultra-performance liquid chromatography and mass spectrometry (UPLC-MS) while others have utilized liquid chromatography in tandem with mass spectrometry (LC-MS) technology exclusively to detect and analyze the substances in chronic gastritis patients' tongue coating. In these studies, some metabolites were differentially expressed between healthy people and patients with chronic gastritis [17, 18]. These differential metabolites may be used as potential markers to monitor the occurrence and development of chronic gastritis. However, these studies only used LC-MS technology. The results produced by using only one detection method may have limitations and cannot fully reflect the metabolites in tongue coating. And, at present, we have not found any reports on metabolomics study of tongue-coating metabolites in patients with GPL that exhibit the damp phlegm pattern.

Therefore, in this study, the ultra-high performance liquid chromatography-Q exactive orbitrap-mass spectroscopy (UHPLC-QE-MS) and gas chromatography-time-of-flight-mass spectroscopy (GC-TOF-MS) were combined in order to detect and analyze the metabolites in the tongue coating of GPL patients with damp phlegm pattern. As a result, the abnormal fluctuation of these metabolites will more clearly show the occurrence and development of GPL damp phlegm pattern.

## 2. Materials and Methods

### 2.1. Samples

In this paper, we used a case-control study to elucidate the composition of tongue-coating-related metabolites in GPL patients with damp phlegm pattern. Sixty patients with GPL were selected from Longhua Hospital, an affiliate of the Shanghai University of TCM for Gastroscopy and pathological examination of gastric mucosa, including 40 cases of damp phlegm pattern and 20 cases of nondamp phlegm pattern. Fifteen teachers and students from the Shanghai University of Traditional Chinese Medicine were selected as a healthy control group. Selected controls did not have a history of stomach disease and were not currently experiencing any degree of stomach discomfort. Their blood cell analysis, blood lipid levels, blood pressure, blood glucose, tumor indicators, renal function, color Doppler ultrasound of neck and abdomen, liver function, chest computed tomography, and barium metal fluoroscopy were all normal. All participants were of Chinese ancestry (self-reported) and were enrolled in the study from December 2018 to October 2019.

Gastroscopy and pathological examination of gastric mucosa were performed immediately after tongue-coating samples were collected. Clinical information of the participants and summary of demographics are provided in Table 1. As shown in Table 1, the highest number of subjects presented with only mild intestinal metaplasia, including 31 damp phlegm pattern patients and 13 nondamp phlegm pattern patients. The number of damp phlegm pattern patients that exhibited either moderate intestinal metaplasia or severe intestinal metaplasia were 7 and 1, respectively. The number of nondamp phlegm pattern patients that exhibited either moderate intestinal metaplasia or severe intestinal metaplasia were 3 and 4, respectively. Finally, only one patient with damp phlegm pattern had mild intestinal metaplasia and mild dysplasia concurrently. In terms of treatment, 10 people did not take the treatment regimen as prescribed due to discomfort (8 cases with damp phlegm pattern and 2 cases with nondamp phlegm pattern), while the other 50 subjects were treated with western medicine or TCM. Among the treated cases, 10 patients with damp phlegm pattern and 5 patients with a nondamp phlegm pattern were treated with western medicine such as proton pump inhibitors alone while 17 patients with damp phlegm pattern and 5 patients with nondamp phlegm pattern were treated with traditional Chinese medicine exclusively. In addition, 5 patients with damp phlegm pattern and 8 patients that exhibited a nondamp phlegm pattern were treated with a combination of traditional Chinese medicine and western medicine (proton pump inhibitors).

### 2.2. Ethics Approval

In this study, all subjects gave written informed consent before collecting samples, and the study was conducted in accordance with the Declaration of Helsinki. In addition, this study was approved by the Ethics Committee of Shanghai University of TCM in December 2018.

### 2.3. Criteria

#### 2.3.1. Diagnostic Criteria

1. Patients were endoscopically examined and biopsies were taken from suspicious lesion sites such as gastric antrum, angle, body, and cardia
2. The histopathological assessment was performed by two experienced pathologists in accordance with the clinical guidelines of “the updated Sydney System” [19]
3. The diagnosis of GPL chronic atrophic gastritis was accompanied by intestinal metaplasia and/or dysplasia [20]

The diagnostic criteria of the damp phlegm pattern in GPL were set according to the “Diagnostics of Traditional Chinese Medicine” [21].

The diagnostic criteria of the damp phlegm pattern in GPL were as follows:

1. Patient felt epigastric fullness or distending pain
2. Patient's feces were not shaped
3. Patients were nauseous and/or vomiting
4. Patient had a bad appetite
5. Patient's tongue had a greasy coating

#### 2.3.2. Inclusion Criteria

The patients met the diagnostic criteria of GPL and the healthy control group had no evidence of systemic organic lesions. In addition, the participants' age range was from 20 to 70 years.

#### 2.3.3. Exclusion Criteria

Patients were not eligible if they had gastric hemorrhaging, a duodenal ulcer, gastroesophageal reflux disease, gastric cancer, gastric ulcers, or other intestinal diseases identified through endoscopic examination as well as any additional systemic diseases. Patients with diagnosed mental illness, pregnant or lactating female subjects, or participants that had lesions of the tongue, mouth, nose, or pharynx within one month prior to the collection of samples were not included in this study. Finally, candidates that had received antibiotics or probiotics within one month prior to sample collection, used tobacco or consumed alcohol, or had a body mass index (BMI) greater than 28 were also excluded [22].

### 2.4. Tongue-Coating Samples Collection

Tongue-coating samples were collected before the participants ate in the morning. Before collection, in order to remove the residues in the mouth, the participants rinsed the mouth with stroke-physiological saline solution 3 times. Then, the collector scraped the tongue-coating samples with a sterile specimen collection swab (CY-98000, iClean, Huachenyang Technology Co., Ltd, CN) at the thick part of the tongue coating 5 times, and put the head of swab with tongue-coating sample into a sterile centrifuge tube. All tongue-coating samples were collected by the same person. Finally, the tongue-coating samples were stored at −80°C.

### 2.5. GC-TOF-MS Metabolomics Processing

The reagents used in GC-TOF-MS experiment are listed in Supplementary Table S1a. The experimental procedures were as follows.

The head of the swab with the tongue-coating sample was moved to a sterile Eppendorf (EP) tube 5 mL and weighed, and 1500 *μ*L of pre-cold extraction mixture with 15 *μ*L of internal standard were added. The sample underwent ultrasonication in the ice water for 30 min. Subsequently, the head of the swab was removed. After centrifugation for 15 minutes at 4°C, the 500 *μ*L supernatant was moved to a fresh tube. Quality control (QC) samples were prepared by combining 100 *μ*L from each sample. T 40 *μ*L of Methoxyamination hydrochloride was added after evaporation in a vacuum concentrator, and the samples were incubated for a half hour at 80°C. After that, 60 *μ*L bis-(trimethylsilyl)-trifluoroacetamide reagent was derivatized for 1.5 hours at 70°C. When the sample was cooled to room temperature, 5 *μ*L of fatty acid methyl esters was added.

GC-TOF-MS detection was performed using a TOF mass spectrometer coupled to an Agilent 7890 gas chromatograph. The system utilized capillary column, 1 *μ*L aliquots of the samples, and helium as carrier gas. The initial temperature was maintained at 50°C, before subsequently was increased to 310°C and held for 6 minutes. The injection temperature was 280°C, the transmission line temperature was 280°C, and the ion source temperature was 250°C. The energy in electron impact mode was −70 ev. After a solvent delay of 6.30 minutes, the mass spectrometry data were obtained in full scan mode, with *m*/*z* range of 50–500. Chroma TOF (V 4.3×, LECO) [23] software was used in order to analyze the original data while the LECO-Fiehn Rtx5 database was utilized to match the mass spectrum and retention index of metabolites. Finally, the peaks with RSD > 30% in QC samples were removed. [24].

### 2.6. UHPLC-QE-MS Metabolomics Processing

The reagents used in UHPLC-QE-MS experiment are listed in Table S1b. The experimental procedures were as follows:

1500 *μ*L of extract solution containing isotopically labeled internal standard mixture was added to the samples. After being vortexed for 30 seconds, the solution was sonicated on ice for 30 minutes. Next, samples were incubated at −40°C for 1 hour and then centrifuged at 4°C for 15 minutes. The supernatant was then isolated and moved to a fresh glass vial for analysis. All the supernatants were mixed to prepare QC samples. The UHPC system using a UPLC BEH Amide column coupled to Q Exactive HFX mass spectrometer was used to detect LC-MS. Twenty-five mmol/L ammonium acetate and 25 ammonia hydroxides in water and acetonitrile were composed in the mobile phase with the elution gradient being used for analysis. The injection volume was 3 *μ*L, the column temperature was 25°C, and the autosampler temperature was 4°C.

The QE HFX mass spectrometer was successful in generating MS/MS spectra under the acquisition software. In this mode, full scan MS spectrum was continuously evaluated by the acquisition software. ProteoWizard was used to convert raw data to mzXML format and peak detection, extraction, alignment, and integration data were obtained through the use of internal programs. Metabolites were annotated used MS2 database (BiotreeDB V2.1), and the cutoff value of annotation was set to 0.3 [25].

### 2.7. Statistical Analysis

The number of peaks, sample names, and standardized peak areas were entered into Simca-p+ 13.0 software for orthogonal projection of principal component analysis (PCA) and orthogonal projection of latent structures-discriminant analysis (OPLS-DA). To further verify the model, displacement experiments were carried out. The *P*-value adjusted with the false discovery rate (FDR) of rank sum test (*P* < 0.05), variable importance in the projection (VIP) of first principal component in OPLS-DA model (VIP > 1), similarity value (SV) of GC-TOF-MS detection (SV > 700) [26], and the significant differences of metabolites between the two groups were determined by MS2 score (MS2 score > 0.6) [27] using UHPLC-QE-MS. The log fold change (FC) values were calculated by comparing the means of metabolites peak areas of the two groups. KEGG pathway analysis was used to search and determine the significant metabolic pathways that were differentially expressed between the experimental and control groups.

## 3. Results

### 3.1. Metabolic Spectrums

Metabolomics of tongue coating in 60 patients with GPL (including 40 cases of damp phlegm pattern and 20 cases of nondamp phlegm pattern) and 15 cases of healthy control group were analyzed by GC-TOF-MS and UHPLC-QE-MS. After quality control by GC-TOF-MS analysis, 533 peaks were obtained in the tongue-coating samples. A total of 9,168 peaks and 4,322 peaks were obtained in tongue-coating samples after quality control of UHPLC-QE-MS positive ion mode and negative ion mode analysis, respectively.

**Figure 1:**
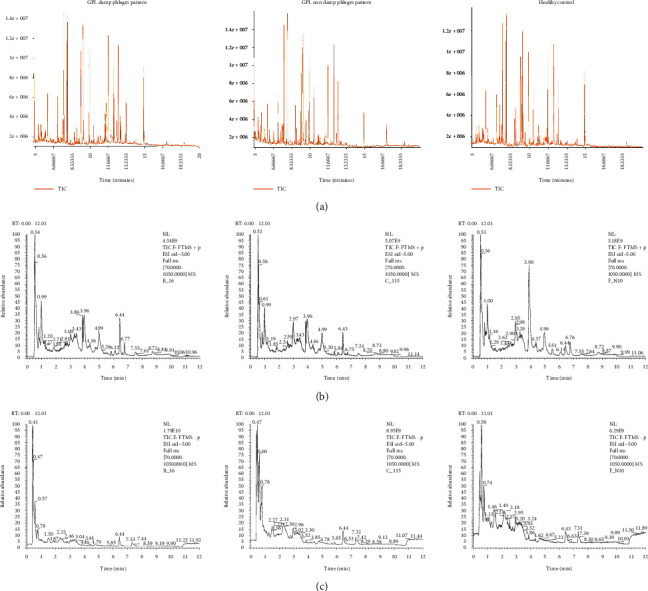
Mass spectrum peaks of GPL damp phlegm pattern group, GPL nondamp phlegm pattern group, and healthy control group. (a) GC-TOF-MS, (b) UHPLC-QE-MS positive ion mode, and (c) UHPLC-QE-MS negative ion mode. As the examples of the GC-TOF-MS and UHPLC-QE-MS spectra, there were some different mass spectrum peaks among the GPL damp phlegm pattern patients, GPL nondamp phlegm pattern patients, and healthy control people.

Examples of GC-TOF-MS and UHPLC-QE-MS spectra of the same person are shown in Figure 1fv. As shown, there were some distinct mass spectrum peaks between the GPL damp phlegm pattern group and the GPL nondamp phlegm pattern group as well as with the healthy control group.

### 3.2. OPLS-DA Score Plots

**Figure 2:**
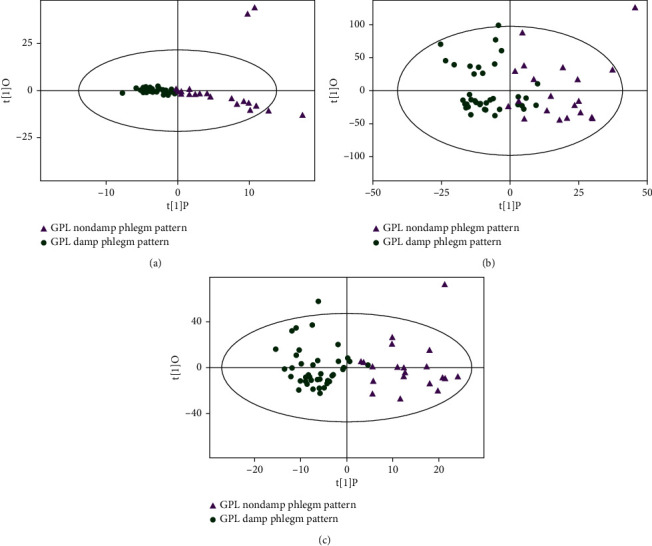
OPLS-DA analysis of metabolomic data of GPL damp phlegm pattern group and nondamp phlegm pattern group. (a) OPLS-DA analysis by GC-TOF-MS, (b) OPLS-DA analysis by UHPLC-QE-MS positive ion mode, and (c) OPLS-DA analysis by UHPLC-QE-MS negative ion mode. The figures of OPLS-DA illustrated that there were significant differences between GPL damp phlegm pattern group and nondamp phlegm pattern group.

**Figure 3:**
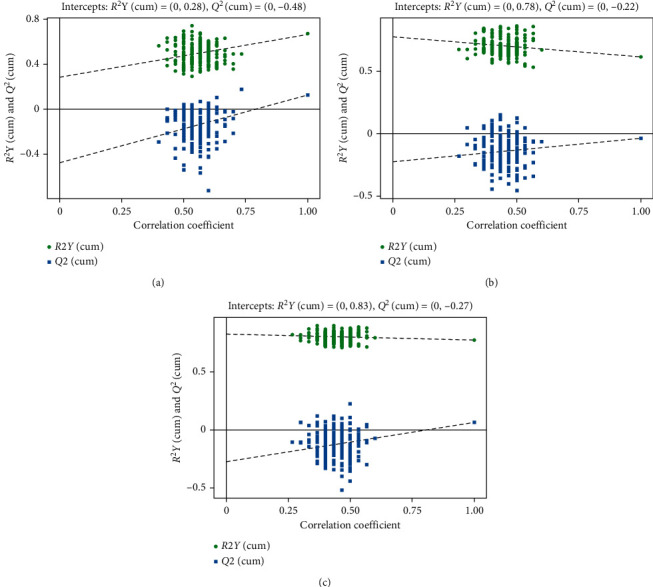
Validation plots of OPLS-DA scores of GPL damp phlegm pattern group and GPL nondamp phlegm pattern group. (a) Validation plots of OPLS-DA scores by GC-TOF-MS, (b) validation plots of OPLS-DA scores by UHPLC-QE-MS positive ion mode, and (c) validation plots of OPLS-DA scores by UHPLC-QE-MS negative ion mode. The figures of validation plots of OPLS-DA scores showed that the models of GPL damp phlegm pattern group and nondamp phlegm pattern group had good robustness as well as had no over fitting phenomenon.

The OPLS-DA score plot of damp phlegm pattern group and nondamp phlegm pattern group showed that the two groups could be well distinguished by GC-TOF-MS and UHPLC-QE-MS negative ion mode. UHPLC-QE-MS positive ion mode detection showed a tendency to distinguish the two groups of samples, but there was a small amount of overlap (Figure 2). The permutation tests of two groups of OPLS-DA models show that all the models of GC-TOF-MS (*R*^2^Y = 0.671, *Q*^2^ = 0.125), UHPLC-QE-MS positive ion mode (*R*^2^Y = 0.617, *Q*^2^ = -0.0357), and UHPLC-QE-MS negative ion mode (*R*^2^Y = 0.775, *Q*^2^ = 0.0659) had good robustness and no overfitting was observed (Figure 3).

**Figure 4:**
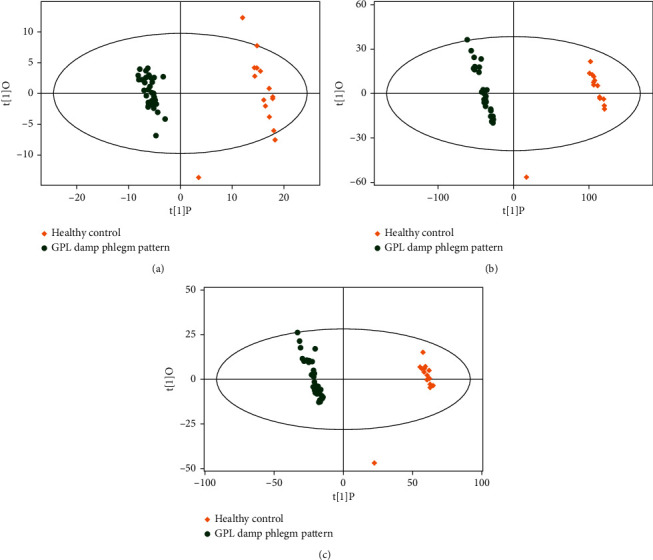
OPLS-DA analysis of metabolomic data of GPL damp phlegm pattern group and healthy control group. (a) OPLS-DA analysis by GC-TOF-MS, (b) OPLS-DA analysis by UHPLC-QE-MS positive ion mode, and (c) OPLS-DA analysis by UHPLC-QE-MS negative ion mode. The figures of OPLS-DA illustrated that there were significant differences between GPL damp phlegm pattern group and healthy control group.

**Figure 5:**
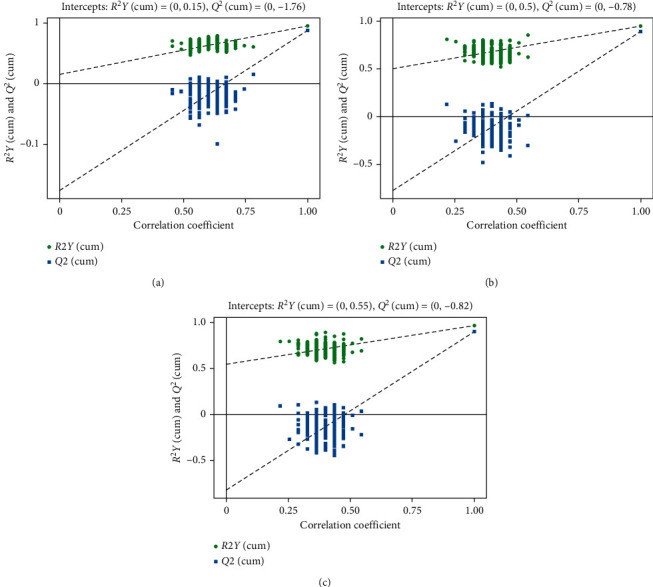
Validation plots of OPLS-DA scores of GPL damp phlegm pattern group and healthy control group. (a) Validation plots of OPLS-DA scores by GC-TOF-MS, (b) validation plots of OPLS-DA scores by UHPLC-QE-MS positive ion mode, and (c) validation plots of OPLS-DA scores by UHPLC-QE-MS negative ion mode. The figures of validation plots of OPLS-DA scores showed that the models of GPL damp phlegm pattern group and healthy control group had good robustness as well as had no over fitting phenomenon.

Similarly, as shown in the OPLS-DA score plot, there was a significant difference between the damp phlegm pattern group and the healthy control group (Figure 4). Permutation test of OPLS-DA models for the two groups showed that all the models of GC-TOF-MS (*R*^2^Y = 0.953, *Q*^2^ = 0.879), UHPLC-QE-MS positive ion mode (*R*^2^Y = 0.951, *Q*^2^ = 0.895), and UHPLC-QE-MS negative ion mode (*R*^2^Y = 0.968, *Q*^2^ = 0.901) had good robustness and no overfitting was observed (Figure 5).

### 3.3. Different Peaks by GC-TOF-MS Analysis

Using the criteria that *P*-value <0.05 and VIP > 1, GC-TOF-MS analysis showed that compared with the nondamp phlegm pattern group, there were 9 different peaks in the damp phlegm pattern group (all the metabolites were downregulated); compared with the healthy control group, there were 33 different peaks in the damp phlegm pattern group, of which 31 peaks increased and 2 decreased.

Using the criteria that similarity SV > 700, GC-TOF-MS analysis showed that compared with the nondamp phlegm pattern group, the phlegm pattern group had 5 different peaks (all metabolites decreased in concentration); compared with the healthy control group, the damp phlegm pattern group had 13 different peaks (all metabolites showed an increase in expression).

### 3.4. Different Peaks by UHPLC-QE-MS Analysis

Using the criteria that *P*-value <0.05 and VIP > 1, UHPLC-QE-MS positive ion mode analysis showed that compared with the nondamp phlegm pattern group, there were 11 different peaks in the damp phlegm pattern group, of which 7 peaks increased and 4 peaks decreased; compared with healthy control group, there were 146 different peaks in the damp phlegm pattern group, of which 142 peaks increased and 4 peaks decreased. UHPLC-QE-MS negative ion mode analysis showed that compared with the nondamp phlegm pattern group, there were 9 different peaks in the damp phlegm pattern group, among which 8 peaks increased and 1 decreased; compared with the healthy control group, there were 23 different peaks in the damp phlegm pattern group (all metabolites increased in concentration).

Using the criteria that MS2 score > 0.6, we align molecular mass data (m/z) of the significantly different peaks with online database KEGG. By UHPLC-QE-MS positive ion mode analysis, when compared with the nondamp phlegm pattern group, the damp phlegm pattern group had 9 different peaks (5 increased, 4 decreased); compared with the healthy control group, the damp phlegm pattern group had 106 different peaks (103 increased, 3 decreased). By UHPLC-QE-MS negative ion mode analysis, compared with the nondamp phlegm pattern group, the damp phlegm pattern group had 8 different peaks (7 increased, 1 decreased); compared with the healthy control group, the damp phlegm pattern group had 106 different peaks (all metabolites increased).

### 3.5. Differential Metabolites Analysis

Among the 156 matched metabolites, the metabolites in the damp phlegm pattern group were mainly divided into 8 categories compared with the nondamp phlegm pattern group. The most abundant metabolites were lipids and lipid-like molecules as well as organic acids and derivatives (each containing 5 metabolites), followed by organic oxygen compounds, organic nitrogen compounds, benzenoids (each containing two metabolites), organoheterocyclic compounds, phenylpropanoids and polyketides, nucleosides, nucleosides, and analogues (each containing one metabolite) (Table 2).

Compared with the healthy controls, the matched metabolites in the damp phlegm pattern group were mainly divided into 10 categories, and the largest metabolite group were lipids and lipid-like molecules (containing 69 metabolites), followed by organoheterocyclic compounds (containing 13 metabolites), organic acids and derivatives (containing 12 metabolites), benzenoids (containing 7 metabolites), organic oxygen compounds, organic nitrogen compounds (each containing 5 metabolites), phenylpropanoids and polyketides (each containing 4 metabolites), homogeneous nonmetal compounds, organosulfon compounds, hydrocarbons (each containing 1 metabolite) (Table 3).

### 3.6. Differential Metabolic Pathways Analysis

According to the annotations of database KEGG on the significantly differential metabolites, following the enrichment analysis and topology analysis, the key pathways with the highest correlation between metabolite differences were found on the basis of both raw *P* and impact values.

In the comparison of damp phlegm pattern group and nondamp phlegm pattern group, GC-TOF-MS analysis generated two pathways (glyoxylate and dicarboxylate metabolism, pentose and glucuronate interconversions), UHPLC-QE-MS positive ion model analysis generated two pathways (primary bile acid biosynthesis and steroid hormone biosynthesis), and UHPLC-QE-MS negative ion mode analysis generated four pathways (arginine and proline metabolism; caffeine metabolism; glycine, serine, and threonine metabolism; purine metabolism). Six key metabolites were involved, including glycolic acid, D-xylitol, cholesterol, L-proline, sarcosine, and xanthine (Table 4).

In the comparison of damp phlegm pattern group and healthy control group, GC-TOF-MS analysis had one pathway named pantothenate and CoA biosynthesis, UHPLC-QE-MS positive ion mode analysis had eight pathways (glycerophospholipid metabolism, sphingolipid metabolism, glycosylphosphatidylinositol (GPI)-anchor biosynthesis, arachidonic acid metabolism, valine, leucine and isoleucine degradation, starch and sucrose metabolism, pentose and glucuronate interconversions, and tyrosine metabolism), UHPLC-QE-MS negative ion mode analysis had one pathway named arachidonic acid metabolism. Ten key metabolites were involved, including pantothenic acid, phosphatidylethanolamine, lysoPC(18 : 1(9Z)), sphinganine 1-phosphate, lactosylceramide, leukotriene D4, 3-methyl-1-hydroxybutyl-ThPP, 3-methoxy-4-hydroxyphenylglycol glucuronide, 5,6-dihydroxyindole, and prostaglandin D2 (Table 5).

## 4. Discussion

GC-TOF-MS and UHPLC-QE-MS have different advantages in the analysis of metabolites. GC-TOF-MS has higher separation ability than UHPLC-QE-MS in the analysis of polar metabolites. Moreover, GC-TOF-MS system has highly repeatable mass spectra, which makes GC-TOF-MS libraries more comprehensive than UHPLC-QE-MS. However, GC-TOF-MS can detect approximately 100 metabolites while UHPLC-QE-MS can detect thousands of metabolites, including semipolar metabolites [28]. Because the composition of metabolites on tongue coating is complex, the combined use of the two detection methods can more comprehensively detect the characteristics of tongue-coating metabolites.

In the metabolomics analysis of damp phlegm pattern tongue-coating samples, lipids and lipid-like molecules were the largest group of differentially expressed metabolites (74 types) when comparing nondamp phlegm pattern patients and healthy controls. It can be seen from Tables 1 and 2 that 72 kinds of lipids and lipid-like molecules were significantly upregulated in the damp phlegm pattern group, and only (3b, 4b, 11b, 14b)-11-ethoxy-3,4-epoxy-14-hydroxy-12-cyathen-15-al 14-xyloside and lactosylceramide (d18 : 1/26 : 0) were significantly downregulated. This result indicates that lipids and lipid-like molecules are the main metabolic disorder differential metabolites of the GPL damp phlegm pattern patient in tongue-coating metabolomics detection. Some studies have found that lipids participate in the development of chronic atrophic gastritis [29]. Chronic gastritis may be related to the global increase in the inflammation state of the body, which was influenced by poor lipid status such as decreased serum high-density lipoprotein (HDL), and the decrease of HDL was closely related to the increased risk of gastric cancer [30, 31]. Some scholars also found that elevated serum-free fatty acids may increase the risk of gastric cancer [32]. In addition, it was also found in experiments on mice that high cholesterol and high-fat diets may increase gastritis incidence in mice [33]. It can be seen that these findings indicate that lipid is related to the occurrence of GPL, but we did not find reports on the relationship between GPL damp phlegm pattern and lipid metabolism. It is worth noting that we found that there is a correlation between damp phlegm pattern and dyslipidemia in other diseases. For example, studies have shown that most patients with hyperlipidemia and atherosclerosis belong to the damp phlegm pattern group, and the level of total cholesterol in hyperlipidemic rats was significantly elevated [34]. Total cholesterol and low-density lipoprotein levels were higher in patients with hypertension-complicated diabetes and damp phlegm pattern [35]. In our previous study, we found that histidine, tryptophan, lysine, and other metabolites exist in chronic renal failure (CRF) and coronary heart disease (CHD) of patients' tongue coating with damp phlegm pattern [11], but these metabolites were not found in the GPL damp phlegm pattern patients' tongue coating, which may be due to the following two reasons. One is that although they are all damp phlegm syndrome, they belong to different diseases, so the metabolites of tongue coating are also different. The second reason is that we only use GC-MS to detect the metabolites of tongue coating in CHD and CRF, which leads to the failure of comprehensive retrieval of the different metabolites of damp phlegm pattern between the two diseases.

According to the metabolic pathway analysis of lipids and lipid-like molecules, six metabolic pathways were involved, including primary bile acid biosynthesis, steroid hormone biosynthesis, glycerophospholipid metabolism, sphingolipid metabolism, glycosylphosphatidylinositol (GPI)-anchor biosynthesis, and arachidonic acid metabolism. The six metabolic pathways contained seven metabolites: cholesterol, phosphatidylethanolamine, lysoPC(18:1(9Z)), sphinganine 1-phosphate, lactosylceramide, leukotriene D4, and prostaglandin D2.

Among them, sphinganine 1-phosphate (S1P) is involved in sphingolipid metabolism. Sphingosine kinase (Sphk) exists in chronic gastritis and gastric cancer cells. Sphk can produce S1P, and many inflammatory reactions are affected by the circulating S1P. The Sphk/S1P axis is an inflammatory mediator in tumor microenvironment and has been identified as a therapeutic target for gastric diseases [38]. Leukotriene D4 and prostaglandin D2 have participated in arachidonic acid metabolism. Arachidonic acid metabolic pathway has an impact on the occurrence and development of gastric cancer [39]. Prostaglandin D2 can inhibit the development of gastric cancer [40]. Leukotriene D4 can aggravate gastric contraction and promote gastric acid secretion [41, 42]. However, gastric pH increases during GPL and gastric cancer [43]. Therefore, leukotriene D4 and prostaglandin D2 as metabolites are considered as new targets for the treatment and prevention of GC [39, 44]. Besides the levels of leukotriene D4 and prostaglandin D2 increased in GPL patients with damp phlegm pattern, which indicates that human body may have certain self-healing function in the process of chronic gastritis developing into gastric cancer. Thus, it can be seen, Sphk/S1P axis, as a target for the treatment of gastric diseases, could be explained from the side that S1P aggravated the development of gastric diseases. The increased expression of leukotriene D4 and prostaglandin D2 indicated that GPL patients had the ability of self-healing in the process of disease.

Among the metabolites of GPL damp phlegm pattern patients, organic acids and derivatives (17 types) and organoheterocyclic compounds (14 types) were the second and third major metabolites. Studies have found that organic acids may be used as a potential metabolic marker of gastric adenocarcinoma, which can be used for the detection, diagnosis, and treatment of gastric adenocarcinoma in the coming years [45]. According to the analysis of metabolic pathways, four metabolites were involved: glycolic acid, pantothenic acid, L-proline, and sarcosine. Among them, pantothenic acid and proline may be potential biomarkers of gastric cancer [46]. Elevated levels of sarcosine may also be associated with the formation of gastric cancer [47]. These studies found that pantothenic acid, proline, and sarcosine all play a role in the development of gastric cancer.

The kinds of other metabolites were fewer; the content of terephthalic acid, L-carnitine, N-undecylbenzenesulfonic acid, kanzonol V, and kanzonol F decreased, while the content of other metabolites increased. Among them the increase of isoxanthopterin may be related to the existence of gastric cancer [48]. Some studies have found that 4-methylcatechol is carcinogenic and can promote gastric cancer and adenocarcinoma [49]. In the experiment of rats, 1,2,4-benzenetriol can increase the thickness of gastric mucosa [50]. 5-ﬂuorouracil (5-Fu), one of the first-line antitumor drugs, can effectively induce the apoptosis of cancer cells while dipyridamole can enhance the cytotoxicity of 5-Fu [51, 52].

According to the annotations of database KEGG on the significantly different metabolites, following the enrichment analysis and topology analysis, on the basis of both raw p and impact values, it was found that glycerophospholipid metabolism was the most important metabolic pathway. The level of phosphatidylethanolamine involved in the synthesis of glycerophospholipid metabolism was significantly increased in group of GPL patients with damp phlegm pattern, and another study has shown that the concentration of phosphatidylethanolamine in the plasma of patients with chronic gastritis increased significantly [52].

However, our research results are still insufficient. Nine GPL patients with damp phlegm pattern were infected with Helicobacter pylori (Hp). Although studies have found that Hp may affect the content of some metabolites in tongue coating of patients with chronic gastritis [53], we previously analyzed the differential metabolites in the tongue coating of 60 patients with GPL compared with healthy people, of which 10 patients were infected with Hp, and the inclusion of these Hp-infected patients did not affect our final screening of differential metabolites [54]. Even so, in future studies, we will still carefully consider the factors of Hp infection.

## 5. Conclusions

In this study, GC-TOF-MS and UHPLC-QE-MS metabolomics technologies were used to detect the metabolites in the tongue coating of GPL patients with damp phlegm pattern for the first time. The results illustrated that there were significant differences in metabolites between GPL patients with damp phlegm pattern and GPL patients with nondamp phlegm pattern as well as healthy people. The types of lipids and lipid-like molecules were the most prominent, involving primary bile acid biosynthesis, steroid hormone biosynthesis, glycerophospholipid metabolism, sphingolipid metabolism, glycosylphosphatidylinositol (GPI)-anchor biosynthesis, and arachidonic acid metabolism. Among them, the most important metabolic pathway was glycerophospholipid metabolism, and the metabolites involved were phosphatidylethanolamine and lysoPC (18:1(9Z)). In addition, organoheterocyclic compounds as well as organic acids and derivatives also contained more kinds of metabolites, which were involved in glyoxylate and dicarboxylate metabolism and pantothenate and CoA biosynthesis as well as arginine and proline metabolism. With further studies, we hope that these different metabolites may be potential diagnostic markers which can be obtained noninvasively for patients with GPL damp phlegm pattern.

## Figures and Tables

**Table 1:** Summary of demographics and clinical information of the participants.

Demographics and clinical information	Damp phlegm pattern group	Nondamp phlegm pattern group	Healthy control group
Sample number	40	20	15
Ratio of male to female	1 : 0.82	1 : 1.22	1 : 1.14
Average age (year)	43.28 ± 14.73	42.9 ± 16.1	34.53 ± 11.40
Number (percentage) of samples diagnosed for less than 10 years	30 (75.00%)	15 (75.00%)	N/A
Number (percentage) of samples diagnosed for 10–20 years	6 (15.00%)	2 (10.00%)	N/A
Number (percentage) of samples diagnosed for 20–30 years	2 (5.00%)	1 (5.00%)	N/A
Number (percentage) of samples diagnosed for 30–40 years	2 (5.00%)	2 (10.00%)	N/A
Number (percentage) of samples only with intestinal metaplasia (mild)	31 (77.50%)	13 (65.00%)	N/A
Number (percentage) of samples only with intestinal metaplasia (moderate)	7 (17.50%)	3 (15.00%)	N/A
Number (percentage) of samples only with intestinal metaplasia (severe)	1 (2.50%)	4 (20.00%)	N/A
Number (percentage) of samples with intestinal metaplasia (mild) and dysplasia (mild)	1 (2.50%)	0 (0.00%)	N/A
Number (percentage) of samples with *Helicobacter pylori* infection	9 (22.50%)	1 (5.00%)	N/A
Number (percentage) of samples untreated	8 (20.00%)	2 (10.00%)	N/A
Number (percentage) of samples only taking western medicine (proton pump inhibitors)	10 (25.00%)	5 (25.00%)	N/A
Number (percentage) of samples only taking traditional Chinese medicine	17 (42.50%)	5 (25.00%)	N/A
Number (percentage) of samples taking western medicine (proton pump inhibitors) and traditional Chinese medicine	5 (12.50%)	8 (40.00%)	N/A

**Table 2:** Identification of significant differential metabolites in tongue coating by comparison of the damp phlegm pattern group and nondamp phlegm pattern group.

Metabolite name	RT (min)	*m*/*z*	Mean damp phlegm pattern	Mean nondamp phlegm pattern	*P*	VIP	Log fold change	Detection method
*Lipids and lipid-like molecules*								
Cholesterol	25.385	369	0.137	0.081	0.028	1.172	0.749	LC+
(3b,4b,11b,14b)-11-ethoxy-3,4-epoxy-14-hydroxy-12-cyathen-15-al 14-xyloside	148.586	495	0.343	0.470	0.040	3.021	−0.453	LC+
PG(18 : 1(11Z)/22 : 5(4Z,7Z,10Z,13Z,16Z))	230.237	824	0.707	0.521	0.048	2.571	0.440	LC+
3a,7b,12a-Trihydroxyoxocholanyl-Glycine	243.238	466	0.648	0.370	0.028	3.020	0.810	LC+
Pantothenol	65.302	204	0.033	0.019	0.015	1.043	0.811	LC−

*Organic acids and derivatives*								
Glycolic acid	5.611	147	0.043	1.045	<0.01	1.104	−4.598	GC
Ustiloxin D	287.091	495	0.041	0.048	0.045	2.236	−0.238	LC+
Leucyl-Valine	398.503	231	0.174	0.095	<0.01	1.230	0.875	LC+
Sarcosine	369.003	88	0.937	0.592	0.045	2.108	2.108	LC−
L-Proline	334.791	114	0.607	0.275	<0.01	1.175	1.175	LC−

*Organic oxygen compounds*								
D-xylitol	9.799	147	0.001	0.051	0.016	1.363	−5.218	GC
L-Iditol	317.933	181	0.023	0.014	<0.01	2.021	0.740	LC−

*Organic nitrogen compounds*								
L-Carnitine	375.368	162	2.626	3.708	0.028	1.077	−0.498	LC+
Dimethyl dialkyl ammonium chloride	65.873	304	0.008	0.002	<0.01	1.158	1.680	LC+

*Benzenoids*								
N-Undecylbenzenesulfonic acid	103.010	313	0.187	0.238	0.031	1.501	−0.342	LC+
Terephthalic acid	372.085	165	1.273	1.524	<0.01	2.378	−0.260	LC−

*Organoheterocyclic compounds*								
Xanthine	233.387	151	4.516	3.253	0.042	1.854	0.473	LC+

*Phenylpropanoids and polyketides*								
Curcumin	98.995	367	0.019	0.013	0.022	1.749	0.493	LC−

*Nucleosides, nucleotides, and analogues*								
Nelarabine	217.309	296	0.034	0.015	<0.01	2.257	1.140	LC−

*Others*								
Ethanol phosphate	6.715	211	0.000	0.007	0.042	1.166	−4.719	GC
6-deoxyglucitol	10.173	117	0.006	0.277	<0.01	1.747	−5.418	GC
Capric acid	8.355	117	0.006	0.236	<0.01	2.048	−5.262	GC

GC, GC-TOF-MS; LC+, UHPLC-QE-MS positive ion; LC−, UHPLC-QE-MS negative ion.

**Table 3:** Identification of significant differential metabolites in tongue coating by comparison of the damp phlegm pattern group and healthy control group.

Metabolite name	RT (min)	*m*/*z*	Mean damp phlegm pattern	Mean healthy control	*P*	VIP	Log fold change	Detection method
*Lipids and lipid-like molecules*								
Foeniculoside VII	71.454	349	0.516	0.057	<0.01	1.411	3.166	LC+
Glycerol tripropanoate	61.345	261	1.152	0.109	<0.01	1.417	3.400	LC+
Solavetivone	115.309	219	0.198	0.014	<0.01	1.081	3.785	LC+
Patchoulenone	61.062	219	3.206	0.278	<0.01	1.238	3.528	LC+
LysoPE(14 : 1(9Z)/0 : 0)	194.563	424	1.679	0.000	<0.01	1.340	14.686	LC+
Gibberellin A79	71.856	365	0.059	0.010	<0.01	1.350	2.615	LC+
Oleic acid	33.290	283	0.246	0.086	<0.01	1.196	1.518	LC+
Dexamethasone	77.202	393	0.241	0.030	<0.01	1.343	3.000	LC+
Leukotriene D4	70.925	497	3.867	1.431	<0.01	1.138	1.434	LC+
Gynosaponin S	73.680	948	0.083	0.013	<0.01	1.251	2.655	LC+
3-O-methylniveusin A	77.445	409	0.040	0.004	<0.01	1.287	3.346	LC+
Hoduloside VII	248.651	932	0.917	0.103	<0.01	1.407	3.153	LC+
L-Acetylcarnitine	328.145	204	0.039	0.025	<0.01	1.092	0.629	LC+
Fucoxanthin	259.139	659	0.081	0.008	<0.01	1.408	3.303	LC+
Citronellyl beta-sophoroside	91.099	481	0.081	0.009	<0.01	1.334	3.169	LC+
N-Cyclopropyl-trans-2-cis-6-nonadienamide	46.068	194	0.175	0.013	<0.01	1.306	3.748	LC+
3-Hydroxyisovalerylcarnitine	57.688	262	0.107	0.010	<0.01	1.405	3.358	LC+
Lyciumoside III	142.803	649	0.026	0.002	<0.01	1.359	3.643	LC+
Fencamfamine	34.439	216	0.275	0.113	<0.01	1.099	1.291	LC+
(3beta,5alpha,9alpha,22E,24R)-5,9-epidioxy-3-hydroxyergosta-7,22-dien-6-one	246.064	443	0.703	0.060	<0.01	1.374	3.547	LC+
Herculin	194.301	252	0.753	0.416	<0.01	1.175	0.858	LC+
Eicosadienoic acid	33.311	309	0.801	0.094	<0.01	1.413	3.096	LC+
3beta-acetoxy-11alpha-methoxy-12-ursen-28-oic acid	193.697	543	0.079	0.009	<0.01	1.217	3.211	LC+
Antibiotic X 14889C	257.759	615	0.086	0.009	<0.01	1.409	3.311	LC+
2-Ethyl-2-hydroxybutyric acid	58.656	133	0.710	0.056	<0.01	1.360	3.670	LC+
Alliospiroside C	120.642	725	0.799	0.113	<0.01	1.386	2.821	LC+
(22E, 24x)-ergosta-4,6,8,22-tetraen-3-one	38.056	393	0.588	0.196	<0.01	1.273	1.585	LC+
3-Methoxy-4-hydroxyphenylglycol glucuronide	293.193	419	0.404	0.045	<0.01	1.411	3.159	LC+
Physalin O	314.512	529	0.128	0.013	<0.01	1.340	3.291	LC+
Fevicordin B 2-gentiobioside	185.565	869	0.274	0.018	<0.01	1.353	3.943	LC+
Desglucocoroloside	220.265	505	0.031	0.008	<0.01	1.074	2.006	LC+
3-Methylglutarylcarnitine	84.851	290	0.270	0.023	<0.01	1.410	3.552	LC+
Smilanippin A	113.780	725	0.602	0.047	<0.01	1.418	3.682	LC+
PS(16 : 1(9Z)/18 : 4(6Z,9Z,12Z,15Z))	207.222	754	0.075	0.007	<0.01	1.407	3.514	LC+
CPA(18 : 0/0 : 0)	278.918	421	0.208	0.020	<0.01	1.416	3.379	LC+
Hericenone E	243.862	595	0.352	0.032	<0.01	1.415	3.474	LC+
Glycerol tributanoate	125.367	303	0.179	0.009	<0.01	1.423	4.287	LC+
Fasciculic acid C	116.186	710	0.444	0.044	<0.01	1.354	3.324	LC+
Hebevinoside I	38.382	809	0.100	0.008	<0.01	1.399	3.557	LC+
Phosphatidylethanolamine	165.695	784	0.121	0.008	<0.01	1.340	3.899	LC+
Lycoperoside D	154.407	740	0.548	0.043	<0.01	1.419	3.659	LC+
Chondrillasterol 3-[glucosyl-(1->4)-glucoside]	97.620	737	0.674	0.131	<0.01	1.338	2.358	LC+
PA(18 : 4(6Z,9Z,12Z,15Z)/14 : 1(9z))	246.799	639	0.178	0.029	<0.01	1.326	2.599	LC+
(24E)-3alpha,15alpha-diacetoxy-23-oxo-7,9(11),24-lanostatrien-26-oic acid	188.646	569	0.359	0.058	<0.01	1.236	2.639	LC+
Physapruin B	227.103	603	0.095	0.021	<0.01	1.146	2.200	LC+
PG(18 : 1(11Z)/22 : 5(4Z,7Z,10Z,13Z,16Z))	230.237	824	0.707	0.015	<0.01	1.430	5.573	LC+
Trihexosylceramide (d18 : 1/12 : 0)	257.583	969	0.362	0.090	<0.01	1.309	2.008	LC+
Traumatic acid	281.345	229	0.507	0.047	<0.01	1.364	3.440	LC+
Lactosylceramide	31.388	1003	0.004	4.397	<0.01	1.316	-10.250	LC+
Sphinganine 1-phosphate	51.580	382	0.149	0.008	<0.01	1.429	4.202	LC+
Budesonide	167.555	431	0.880	0.012	<0.01	1.228	6.183	LC+
3-Benzoyloxy-11-oxo-12-ursen-28-oic acid	34.377	575	2.136	0.156	<0.01	1.422	3.776	LC+
3a,7b,12a-Trihydroxyoxocholanyl-Glycine	243.238	466	0.648	0.047	<0.01	1.051	3.788	LC+
Momordicoside K	69.766	649	0.691	0.161	<0.01	1.340	2.100	LC+
PI(22 : 5(4Z,7Z,10Z,13Z,16Z)/16 : 0)	244.507	886	0.141	0.010	<0.01	1.338	3.842	LC+
Vinaginsenoside R3	84.853	932	0.486	0.035	<0.01	1.418	3.811	LC+
PA(18 : 4(6Z,9Z,12Z,15Z)/18 : 4(6Z,9Z,12Z,15Z))	144.619	689	0.018	0.001	<0.01	1.185	3.617	LC+
Dodecanoic acid	49.522	199	10.480	5.041	<0.01	1.467	1.056	LC−
Hydroxyisocaproic acid	251.772	131	3.168	0.297	<0.01	1.428	3.414	LC−
5,15-DiHETE	108.697	335	0.119	0.002	<0.01	1.741	5.644	LC−
Adipic acid	394.038	145	7.034	1.213	<0.01	1.584	2.536	LC−
13S-hydroxyoctadecadienoic acid	50.007	295	0.642	0.230	<0.01	1.585	1.481	LC−
Prostaglandin D2	46.555	351	1.268	0.105	<0.01	1.190	3.601	LC−
9,10-DHOME	81.276	313	0.120	0.008	<0.01	1.607	3.946	LC−
Azelaic acid	359.713	187	0.188	0.010	<0.01	1.787	4.170	LC−
(10E,12Z)-(9S)-9-hydroperoxyoctadeca-10,12-dienoic acid	60.715	311	0.120	0.005	<0.01	1.703	4.569	LC−-
6beta-hydroxyasiatic acid	51.604	503	0.135	0.012	<0.01	1.762	3.463	LC−
Fasciculol C	257.943	553	0.006	0.001	<0.01	1.354	3.406	LC−
Traumatic acid	139.932	227	0.230	0.022	<0.01	1.608	3.369	LC−

*Organic acids and derivatives*								
Pantothenic acid	11.358	103	0.014	0.002	<0.01	1.118	2.536	GC
Bortezomib	285.056	407	0.077	0.006	<0.01	1.414	3.773	LC+
Ustiloxin D	287.091	495	0.041	0.003	<0.01	1.412	3.737	LC+
N,N-Dimethylformamide	300.102	74	0.143	0.085	<0.01	1.035	0.743	LC+
Oseltamivir	314.576	313	0.143	0.015	<0.01	1.322	3.249	LC+
Arginyl-arginine	296.631	331	2.122	0.170	<0.01	1.421	3.643	LC+
Arginyl-histidine	166.723	312	0.412	0.030	<0.01	1.419	3.784	LC+
Dihydro-3-(2-octenyl)-2,5-furandione	281.391	211	0.131	0.017	<0.01	1.243	2.963	LC+
N-Acetylleucine	265.067	174	0.140	0.010	<0.01	1.352	3.829	LC+
L-cis-3-amino-2-pyrrolidinecarboxylic acid	75.782	131	0.027	0.002	<0.01	1.340	3.693	LC+
L-Homocysteic acid	124.120	182	0.127	0.008	<0.01	1.786	3.970	LC−
3-Hydroxycapric acid	83.306	187	0.241	0.088	<0.01	1.450	1.448	LC−

*Organic oxygen compounds*								
Leonuriside A	372.037	333	0.012	0.001	<0.01	1.429	4.365	LC+
Kanamycin	205.447	485	0.036	0.004	<0.01	1.372	3.253	LC+
Guanadrel sulfate	79.840	214	3.912	0.331	<0.01	1.418	3.562	LC+
Trimethylaminoacetone	206.235	116	0.113	0.007	<0.01	1.228	4.102	LC+
4-Ipomeanol	72.941	167	0.311	0.024	<0.01	1.004	3.699	LC−

*Benzenoids*								
Dictagymnin	60.998	203	0.068	0.005	<0.01	1.081	3.698	LC+
4'-hydroxyfenoprofen glucuronide	281.454	435	0.089	0.007	<0.01	1.401	3.626	LC+
Methoxamine	410.726	212	0.037	0.004	<0.01	1.302	3.038	LC+
N-undecylbenzenesulfonic acid	103.010	313	0.187	0.092	<0.01	1.133	1.031	LC+
Salmeterol	57.091	416	0.468	0.044	<0.01	1.419	3.415	LC+
Esmolol	175.513	296	0.168	0.015	<0.01	1.345	3.498	LC+
(Â±)-2-(1-methylpropyl)-4,6-dinitrophenol	31.378	239	2.850	1.286	<0.01	1.073	1.148	LC−

*Organoheterocyclic compounds*								
Pirenzepine	132.148	352	0.433	0.047	<0.01	1.403	3.206	LC+
Neferine	264.617	625	0.845	0.118	<0.01	1.390	2.841	LC+
Pyrimidine	112.505	81	0.097	0.008	<0.01	1.416	3.566	LC+
5,6-Dihydroxyindole	45.066	150	0.256	0.031	<0.01	1.226	3.029	LC+
2-Pyrrolidineacetic acid	64.058	130	0.684	0.340	<0.01	1.165	1.009	LC+
Alpha-carboxy-delta-decalactone	125.959	215	0.085	0.004	<0.01	1.413	4.275	LC+
I-Urobilin	174.113	591	0.102	0.006	<0.01	1.413	4.117	LC+
4-Ethyl-5-methyl-2-(1-methylethyl)oxazole	244.532	154	0.657	0.081	<0.01	1.249	3.023	LC+
Garcimangosone C	282.076	413	0.893	0.071	<0.01	1.405	3.654	LC+
8-Hydroxycarteolol	116.908	309	0.164	0.018	<0.01	1.415	3.203	LC+
3-Methyl-1-hydroxybutyl-ThPP	281.408	511	0.040	0.003	<0.01	1.394	3.675	LC+
Azaspiracid 3	109.997	828	0.089	0.034	<0.01	1.000	1.385	LC+
3'-N-acetyl-4'-O-(9-octadecenoyl)fusarochromanone	328.728	599	0.027	0.004	<0.01	1.080	2.884	LC+

*Organic nitrogen compounds*								
1-Butylamine	268.847	74	11.933	0.834	<0.01	1.360	3.839	LC+
Nervonyl carnitine	237.324	102	659.917	66.315	<0.01	1.342	3.315	LC+
Isobutylpropylamine	231.650	116	0.444	0.041	<0.01	1.417	3.420	LC+
2-Diethylaminoethanol	300.009	118	195.976	13.198	<0.01	1.424	3.892	LC+
Dipyridamole	47.761	253	0.298	0.016	<0.01	1.426	4.263	LC+

*Phenylpropanoids and polyketides*								
Kanzonol V	244.573	377	0.005	0.457	<0.01	1.096	−6.613	LC+
Sofalcone	285.882	451	0.063	0.005	<0.01	1.415	3.788	LC+
Kanzonol F	247.943	421	0.003	0.360	<0.01	1.135	-7.002	LC+
Kanzonol I	83.271	437	0.060	0.000	<0.01	1.051	9.876	LC+

*Homogeneous nonmetal compounds*								
Phosphoric acid	32.551	99	0.643	0.052	<0.01	1.246	3.632	LC+

*Organosulfur compounds*								
(Â±)-2-pentanethiol	32.769	105	0.397	0.080	<0.01	1.089	2.302	LC+

*Hydrocarbons*								
1-Methyl-1,3-cyclohexadiene	32.571	95	0.477	0.093	<0.01	1.182	2.356	LC+

*Others*								
6-deoxyglucitol	10.173	117	0.006	0.001	<0.01	2.220	2.432	GC
1,2,4-benzenetriol	9.344	124	0.213	0.015	<0.01	2.240	3.856	GC
Isoxanthopterin	12.091	100	0.002	0.001	<0.01	1.598	1.130	GC
Lactobionic acid 2	14.932	147	0.032	0.013	<0.01	1.342	1.289	GC
2,4-dichloro-1-(2-chloroethenyl)-benzene	7.925	170	0.476	0.034	<0.01	2.341	3.823	GC
Asparagine dehydrated	8.628	110	0.011	0.002	<0.01	2.212	2.434	GC
4-Hydroxybenzoate	9.423	110	2.458	0.161	<0.01	2.343	3.933	GC
Conduritol-beta-expoxide	11.541	103	0.174	0.019	<0.01	2.231	3.190	GC
Methylmaleic acid	7.923	170	0.477	0.034	<0.01	2.341	3.826	GC
4-Methylcatechol	7.928	170	0.452	0.034	<0.01	2.148	3.749	GC
Beta-mannosylglycerate	12.271	204	0.000	0.000	<0.01	1.350	0.739	GC
Uridine minor	13.354	145	0.066	0.007	<0.01	2.318	3.263	GC
(10S,11R)-pterosin C 4-glucoside	282.232	397	16.094	1.266	<0.01	1.331	3.669	LC+
PC(P-18 : 1(9Z)/16 : 0)	36.173	745	0.693	0.070	<0.01	1.416	3.299	LC+
LysoPC(18:1(9Z))	210.778	544	0.019	0.001	<0.01	1.004	4.867	LC+
PC(P-16 : 0/18 : 4(6Z,9Z,12Z,15Z))	153.493	739	0.048	0.005	<0.01	1.200	3.387	LC+

GC: GC-TOF-MS; LC+: UHPLC-QE-MS positive ion; LC−: UHPLC-QE-MS negative ion.

**Table 4:** Key metabolic pathways identified from the significant differential metabolites in tongue coating between the damp phlegm pattern group and nondamp phlegm pattern group.

Pathway	Raw *P*	Impact	Significantly different metabolites
*GC-TOF-MS metabolomics analysis*			
Glyoxylate and dicarboxylate metabolism	0.041	0.007	Glycolic acid
Pentose and glucuronate interconversions	0.044	0.032	D-Xylitol

*UHPLC-QE-MS positive ion mode metabolomics analysis*			
Primary bile acid biosynthesis	0.057	0.055	Cholesterol
Steroid hormone biosynthesis	0.118	0.004	Cholesterol

*UHPLC-QE-MS negative ion mode metabolomics analysis*			
Arginine and proline metabolism	0.025	0.109	L-Proline; sarcosine
Caffeine metabolism	0.068	0.031	Xanthine
Glycine, serine and threonine metabolism	0.149	0.050	Sarcosine
Purine metabolism	0.268	0.036	Xanthine

**Table 5:** Key metabolic pathways identified from the significant differential metabolites in tongue coating between the damp phlegm pattern group and healthy control group.

Pathway	Raw *P*	Impact	Significantly different metabolites
*GC-TOF-MS metabolomics analysis*			
Pantothenate and CoA biosynthesis	0.044	0.180	Pantothenic acid

*UHPLC-QE-MS positive ion mode metabolomics analysis*			
Glycerophospholipid metabolism	0.018	0.231	Phosphatidylethanolamine; LysoPC(18:1(9Z))
Sphingolipid metabolism	0.050	0.017	Sphinganine 1-phosphate; lactosylceramide
Glycosylphosphatidylinositol(GPI)-anchor biosynthesis	0.186	0.044	Phosphatidylethanolamine
Arachidonic acid metabolism	0.227	0.026	Leukotriene D4
Valine, leucine and isoleucine degradation	0.446	0.035	3-Methyl-1-hydroxybutyl-ThPP
Starch and sucrose metabolism	0.523	0.013	3-Methoxy-4-hydroxyphenylglycol glucuronide
Pentose and glucuronate interconversions	0.544	0.009	3-Methoxy-4-hydroxyphenylglycol glucuronide
Tyrosine metabolism	0.677	0.007	5,6-Dihydroxyindole

*UHPLC-QE-MS negative ion mode metabolomics analysis*			
Arachidonic acid metabolism	0.042	0.034	Prostaglandin D2

## Data Availability

The Ethics Committee of Shanghai University of Traditional Chinese Medicine limited the measurement data used to support the results of this study in order to protect the privacy of patients. For researchers who meet the criteria for obtaining confidential data, the data of this study can be obtained from Yiming Hao (e-mail: hymjj888@163.com).

## Supplementary Materials

Supplementary Materials Table S1 shows the reagents used in GC-TOF-MS and UHPLC-QE-MS experiments.
