# Supplementary material for: Metabolite Characteristics in Tongue Coating from Damp Phlegm Pattern in Patients with Gastric Precancerous Lesion
Source: Evid Based Complement Alternat Med. 2021 Jun 2;2021:5515325. doi: 10.1155/2021/5515325 (PMC8189775; doi:10.1155/2021/5515325)
Supplement: Supplementary Materials — Table S1 shows the reagents used in GC-TOF-MS and UHPLC-QE-MS experiments. [file 5515325.f1.docx]

**Table S1. Reagents used in the experiments**

| Reagents | Manufacturer |
| --- | --- |
| ***a. Reagents used in GC-TOF-MS experiment*** | |
| Methanol | CNW Technologies, Germany |
| Chloroform | Adamas, Switzerland |
| Pyridine | Adamas, Switzerland |
| Methoxy amination hydrochloride | Tokyo Chemical Industry, Japan |
| Adonitol | Sigma-Aldrich, USA |
| Bis-(trimethylsilyl)-trifluoroacetamide (with 1% trimethylchlorosilane, v/v) | Regis Technologies, USA |
| Fatty acid methyl esters | Dr. Ehrenstorfer, Germany |
| ***b. Reagents used in UHPLC-QE-MS experiment*** | |
| Methanol | CNW Technologies, Germany |
| Acetonitrile | CNW Technologies, Germany |
| Ammonium acetate | CNW Technologies, Germany |
| Ammonium hydroxide | CNW Technologies, Germany |
